# Designing the matrix: extracellular matrix-informed strategies for bioengineered cancer models

**DOI:** 10.3389/fbioe.2026.1894741

**Published:** 2026-08-24

**Authors:** Nathalie Bock, Anu Thomas Koikalethu, Luke Hipwood, Minne Dekker, Brooke Lundon, Sugandha Bhatia, Jacqui A. McGovern

**Affiliations:** 1 Max Planck Queensland Centre for the Materials Science of Extracellular Matrices (MPQC), Brisbane, QLD, Australia; 2 School of Biomedical Sciences, Faculty of Health, Queensland University of Technology (QUT) at the Translational Research Institute (TRI), Centre for Biomedical Technologies (CBT), Woolloongabba, QLD, Australia; 3 Gelomics Pty Ltd., Brisbane, QLD, Australia; 4 ARC Training Centre for Cell and Tissue Engineering Technologies, QUT, Brisbane, QLD, Australia

**Keywords:** 3D cancer model, biomaterials, drug response, ECM remodelling, extracellular matrix, hydrogels, matrix mechanics, tumour microenvironment

## Abstract

Bioengineered three-dimensional (3D) cancer models have improved the replication of tumour architecture and microenvironmental complexity, yet their predictive value and reproducibility remain inconsistent. A key reason is that extracellular matrix (ECM) context is often treated as a background culture condition rather than as an explicit experimental variable. Cancer cells respond not only to dimensionality, but also to coupled biochemical, structural, mechanical and dynamic matrix cues, including ligand presentation, fibre architecture, porosity, stiffness, viscoelasticity, degradability and remodelling. In this mini-review, we examine ECM-mimicking cancer models through a materials science-informed lens and argue that matrix selection should be guided by model purpose rather than perceived physiological complexity alone. We discuss tissue-derived matrices for preserving tissue-specific and patient-relevant cues, natural and semi-synthetic hydrogels for balancing biological context with usability, synthetic matrices for mechanistic dissection, transport-aware systems for drug-response studies and dynamic hydrogels for modelling time-dependent matrix remodelling. We propose that ECM mimicry should move from a descriptive label to a hypothesis-driven design principle. Making matrix design explicit will improve comparison across models, reduce ambiguity in interpreting cancer phenotypes and strengthen the mechanistic and translational relevance of *in vitro* cancer systems.

## 1 Introduction

Traditional two-dimensional (2D) culture systems have been central to cancer research, but they do not reproduce the tissue architecture and three-dimensional (3D) cell–matrix interactions that shape tumour behaviour (Rauner et al., 2025; Muncie and Weaver, 2018). They also poorly capture spatial gradients, matrix-imposed transport constraints and biophysical cues that influence invasion, dormancy and therapeutic response (Stylianopoulos et al., 2018; Chitty and Cox, 2025). Therefore, 3D cancer models, including spheroids (Bruns et al., 2023; Xu et al., 2024), organoids (Bock et al., 2023; Nam et al., 2024; Song et al., 2024), tumour-on-chip systems (Wu et al., 2026) and matrix-embedded cultures (Goodarzi and Rao, 2024; Hipwood et al., 2026a; Sinha et al., 2023), have become important tools for preserving aspects of tissue organisation and microenvironmental heterogeneity (Rauner et al., 2025; Herrmann et al., 2014; Zhang et al., 2026; Bray et al., 2019). Yet dimensionality alone does not confer physiological relevance (Fischbach et al., 2009).

The ECM is not merely structural support, but a dynamic, tissue-specific regulator of cancer behaviour (Naba, 2024; Pickup et al., 2014). Cells continually remodel the ECM through deposition of nascent matrix, proteolytic degradation and secretion of matrix-modifying enzymes, while the ECM regulates adhesion, migration, differentiation, survival and signalling (Muncie and Weaver, 2018). In cancer, this reciprocal regulation becomes deregulated as tumour and stromal cells alter ECM composition, collagen cross-linking, fibre organisation, stiffness, viscoelasticity and matrix-sequestered growth factor signalling (Pickup et al., 2014). These changes support invasion, immune evasion, dormancy, metastatic niche formation and therapy resistance (Chitty and Cox, 2025; Hanahan, 2026). For *in vitro* modelling, this means that matrix context cannot be treated as a passive scaffold, as interdependent differences in ECM composition, architecture, mechanics or remodelling capacity can alter cancer cell behaviour across otherwise similar 3D platforms (Hipwood et al., 2026a; Bhatia et al., 2026).

Here, we use “bioengineered 3D cancer models” to describe *in vitro* systems in which cancer cells, spheroids, organoids or patient-derived tumour cells are cultured within controlled 3D environments designed to reproduce selected features of the tumour microenvironment (Rauner et al., 2025). Within this category, ECM-mimicking cancer models aim to recapitulate selected biochemical, structural, mechanical or remodelling properties of the ECM using natural, synthetic, semi-synthetic or tissue-derived materials (Bock et al., 2023; Shimpi and Fischbach, 2021). These models differ in the biochemical cues they present, including adhesive ligands and basement membrane or interstitial matrix proteins (Bock et al., 2023; Sui et al., 2025). They also vary in fibre architecture and degradability (Enriquez Martinez et al., 2025; Sievers et al., 2023), dynamic mechanical properties such as viscoelasticity (Tolentino et al., 2025a; Fan et al., 2024), and transport properties that influence diffusion of nutrients, drugs and soluble factors (Stylianopoulos et al., 2018).

Several studies illustrate this point. In 3D-bioprinted tumour spheroid models spanning cervical, liver and lung cancer cell lines, doxorubicin EC50 values were more than two-fold higher than in matched 2D cultures, with resistance linked to multidrug-resistance transporter phenotype and partially reversed by ABC transporter inhibition (Hong et al., 2025). In patient-derived osteosarcoma models, matrix-free and Matrigel®-based 3D cultures preserved patient-specific heterogeneity but produced different transcriptional states linked to ECM production, stemness and plasticity (Bhatia et al., 2026). Hipwood et al. showed that stiffness-matched LungMA, GelMA and basement membrane extract models produced distinct A549 morphologies and chemotherapy responses, highlighting that matrix composition and architecture can alter model behaviour even when dimensionality and stiffness are controlled (Hipwood et al., 2026a).

Together, these studies highlight a broader issue in ECM-mimicking cancer models: cells respond to coupled biochemical, structural and biophysical matrix cues, rather than to dimensionality, stiffness or composition in isolation (Muncie and Weaver, 2018; Sui et al., 2025). Incomplete characterisation of biophysical properties, including viscoelasticity, porosity, microstructure and transport-related properties, may therefore contribute to divergent outcomes across 3D platforms (Stylianopoulos et al., 2018; Sui et al., 2025; Cameron et al., 2022). The key design challenge is therefore to define which ECM features should be reproduced, simplified or measured so that ECM-mimicking cancer models capture the matrix features most relevant to the biological process being studied (Lutolf and Hubbell, 2005; Tibbitt and Anseth, 2009).

In this mini-review, we argue that ECM selection and characterisation should be treated as deliberate design choices rather than background culture conditions. Instead of assuming a model is more physiologically relevant because it offers some matrix-supporting features, ECM-mimicking cancer models should be evaluated by how well closely matrix features align with their intended purpose.

## 2 Which ECM features drive model divergence?

**FIGURE 1:**
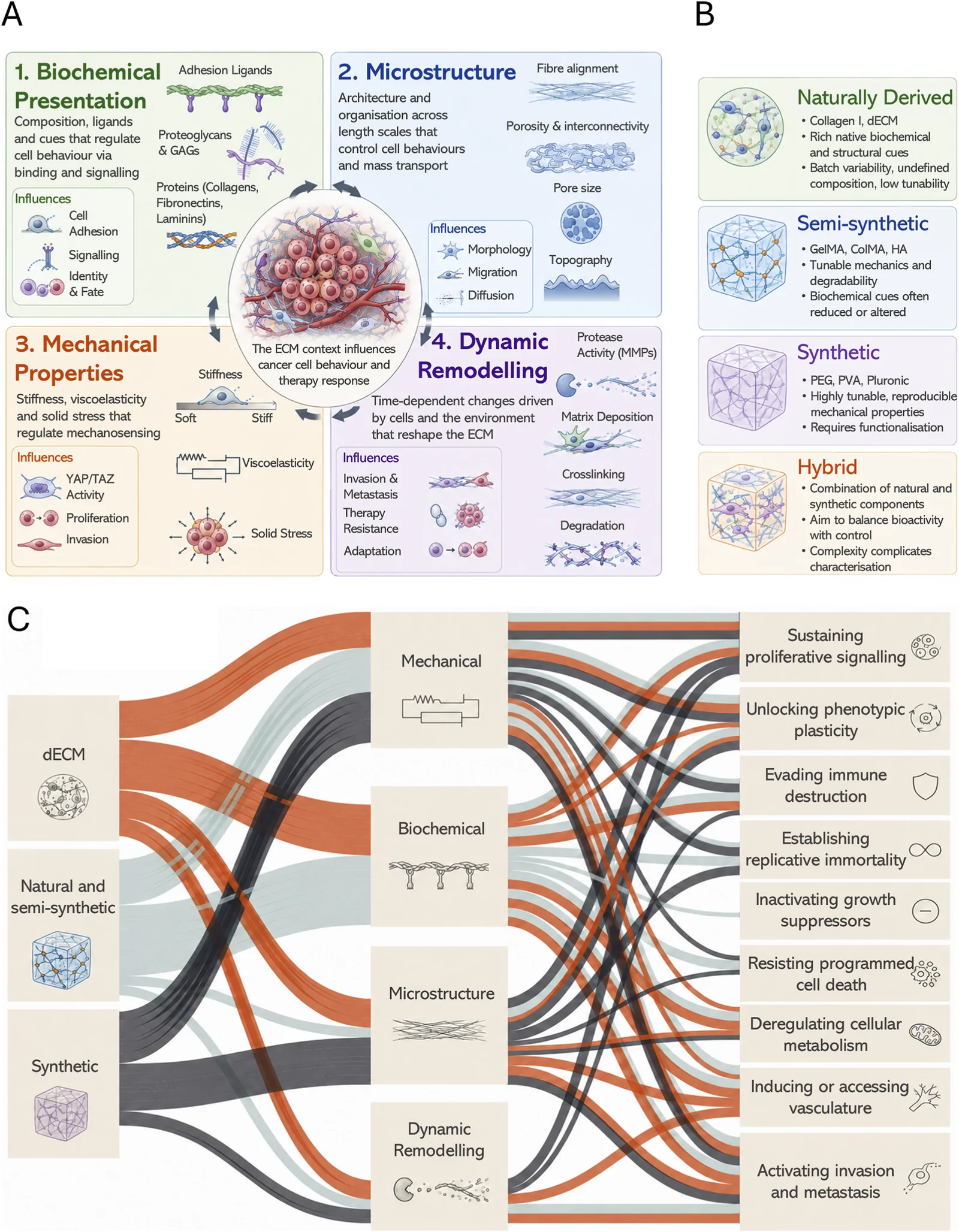
ECM-mimicking biomaterials and interacting matrix properties in cancer design and modelling. **(A)** Overview of four interacting ECM property categories: biochemical presentation, microstructure, mechanical properties and dynamic remodelling, and their influence on cancer cell behaviour and therapy response. **(B)** Common ECM-mimicking biomaterial classes used in 3D cancer models, including naturally derived, semi-synthetic, synthetic and hybrid/composite approaches. **(C)** ECM model design map linking biomaterial classes to matrix properties and cancer hallmark-related behaviours. Link colours indicate biomaterial categories: dECM (orange (Wu et al., 2026; Kim et al., 2022; Choi et al., 2025; Ahn et al., 2023; Chen et al., 2022; Chen et al., 2024; Didwischus et al., 2024; Fok et al., 2023; He et al., 2026; Wang et al., 2023; Weng et al., 2024)), natural and semi-synthetic materials (blue (Goodarzi and Rao, 2024; Cameron et al., 2022; Cao et al., 2021; Chang and Lin, 2021; Fisher et al., 2018; Gu and Mooney, 2016; Isik et al., 2022; Kaemmerer et al., 2014; Mette et al., 2020; Liu et al., 2024; Luo et al., 2021; Molley et al., 2023; Northcutt et al., 2019)), and synthetic materials (grey (Below et al., 2021; Cook et al., 2017; Foroni et al., 2013; Medina et al., 2019; Balachander et al., 2015; Odabasi et al., 2026; Polonio-Alcalá et al., 2019; Pradhan and Slater, 2019; Singh et al., 2015; Wang et al., 2014; Wang et al., 2022; Yaginuma et al., 2020; Gill et al., 2012; Feng et al., 2013; Rauner et al., 2025; Muncie and Weaver, 2018; Stylianopoulos et al., 2018; Chitty and Cox, 2025; Bruns et al., 2023; Xu et al., 2024; Bock et al., 2023; Nam et al., 2024; Song et al., 2024; Wu et al., 2026; Goodarzi and Rao, 2024; Hipwood et al., 2026a; Sinha et al., 2023; Herrmann et al., 2014)). Central nodes denote key interacting ECM properties, while nodes on the right depict ECM–cancer functional behaviours associated with established cancer hallmarks (Hanahan, 2026). Link thickness represents a literature-derived weighted score indicating the relative strength of reported associations, classified as weak (1) or strong (2) based on whether the study reported an indirect/contextual link or a direct experimentally supported relationship between the biomaterial category, ECM property and cancer behaviour. The Sankey diagram was generated in RStudio using R v4.5.1.

Cancer cell behaviour is influenced by multiple interdependent ECM features (Figure 1A) (Muncie and Weaver, 2018; Naba, 2024; Pickup et al., 2014). From a materials science perspective, these can be grouped into four interacting categories: (i) biochemical presentation, including ECM composition, adhesive motifs such as Arg-Gly-Asp (RGD), growth factor-binding sites and proteolytic cleavage sites; (ii) microstructure, including fibre alignment, mesh size and topography; (iii) mechanics, including elastic modulus and stress relaxation; and (iv) dynamic remodelling, including cell-mediated degradation, nascent ECM deposition and crosslinking (Bock et al., 2024; Bonnans et al., 2014).

This classification helps define what a model aims to reproduce, but model divergence arises because cells experience these ECM features together. Differences in degradability and remodelling capacity can shift invasion strategies, feedback on biomechanics and alter ligand or matrix-sequestered factor presentation. Koorman et al. showed that spatial collagen stiffening promotes collective breast cancer invasion by reinforcing ECM fibre alignment, linking architecture and mechanics to invasion mode (Koorman et al., 2022). Pourmostafa et al. further showed that, in 3D hydrogel microtissues, ECM biophysical properties, particularly stiffness and porosity, were stronger determinants of MDA-MB-231 cell morphology than biochemical composition (Pourmostafa et al., 2026). These effects also extend to immune cells, key cells within the tumour microenvironment. Variations in collagen density have been associated with reduced CD8^+^ T-cell infiltration in breast tumours and high density collagen directly suppressed tumour-infiltrating T cell proliferation and cytotoxicity in 3D collagen matrices (Kuczek et al., 2019). Thus, cellular phenotypes cannot be inferred from bulk mechanics or composition alone; hydrogel porosity also depends on network formulation and microstructure, with implications for cell confinement and transport (Tolentino et al., 2025b).

Viscoelasticity is a clear but under-characterised example of ECM interdependence because, unlike bulk stiffness, it is less routinely reported and requires time-dependent mechanical testing. It describes behaviours such as stress relaxation and creep, which influence how cells exert, dissipate and respond to forces within the matrix (Cameron et al., 2022; Courbot and Elosegui-Artola, 2025). Martinez-Garcia et al. showed that organ-derived ECM hydrogel stress relaxation and gelation vary with tissue-specific composition and ultrastructure (Martinez‐garcia et al., 2021). In glioblastoma models, Sinha et al. used PEG-collagen-I interpenetrating network hydrogels to tune stress relaxation while maintaining brain-mimicking stiffness, ligand density and diffusion (Sinha et al., 2023). Faster stress relaxation promoted cell spreading, migration, stem-like marker expression, proliferation and altered drug sensitivity. Fan et al. showed that collagen architectural changes increased liver ECM viscoelasticity without increasing stiffness, promoting hepatocellular carcinoma proliferation and invasion through integrin-β1–tensin-1–YAP signalling (Fan et al., 2024). Lin et al. further showed that viscoelastic hydrogels promoted CAF spreading and enhanced pancreatic cancer cell proliferation, spreading and EMT-associated secretory changes (Lin et al., 2023). These studies argue that viscoelasticity should be reported when comparing ECM-mimicking models or interpreting mechanically driven phenotypes.

Transport provides another practical example of how coupled ECM properties drive divergence. Diffusion of oxygen, nutrients, soluble factors and therapeutics is governed by matrix architecture, porosity, density and hydration (Stylianopoulos et al., 2018; Salavati et al., 2023). Xu et al. combined spatial drug uptake and growth-rate imaging to show that lapatinib penetration and treatment response varied across A549 spheroid layers, with higher drug accumulation in outer regions and distinct growth inhibition patterns toward the spheroid centre (Xu et al., 2024). In colorectal cancer cells embedded in collagen-I-based colon dECM hydrogels, D’Angelo et al. showed that increasing matrix concentration increased stiffness, reduced porosity, delayed doxorubicin diffusion and uptake, and increased 5-FU resistance (D’Angelo et al., 2026). Transport-related properties should therefore be characterised when ECM-mimicking models are used to compare therapeutic responses or infer matrix-mediated resistance (Stylianopoulos et al., 2018; D’Angelo et al., 2026).

## 3 Choosing ECM-mimicking cancer models by purpose

ECM-mimicking cancer models span tissue-derived matrices that retain broad biochemical complexity (D’Angelo et al., 2026; Golebiowska et al., 2024; Milton et al., 2024; Barros da Silva et al., 2024), natural and semi-synthetic hydrogels that incorporate selected ECM-like features (Tibbitt and Anseth, 2009; Alsharabasy and Pandit, 2024; Loessner et al., 2016; Bessot et al., 2025; Joshi et al., 2026), and synthetic systems designed to isolate variables such as ligand presentation, degradability, stiffness or stress relaxation (Figure 1B) (Bock et al., 2023; Katz and West, 2022; Ligorio and Mata, 2023). As summarised in Figure 1, these platforms are best viewed as design strategies rather than as a hierarchy of physiological relevance. The appropriate choice depends on model purpose (Figure 1C), including tissue-specific or patient-relevant fidelity, biological context with usability, mechanistic dissection of defined ECM cues, transport-sensitive drug-response testing, or modelling dynamic ECM remodelling. Table 1 summarises the corresponding ECM strategies, characterisation priorities and trade-offs.

**TABLE 1:** Fit-for-purpose selection of ECM-mimicking cancer models.

Model purpose	Suitable ECM strategy	Prioritise/characterise	Main trade-off
Tissue-specific fidelity/patient relevance	Tissue-derived matrices, dECM, organ-specific ECM hydrogels, BME/Matrigel® (Nam et al., 2024; Song et al., 2024; Hipwood et al., 2026a; D’Angelo et al., 2026; Golebiowska et al., 2024; Milton et al., 2024; Barros da Silva et al., 2024; Hipwood et al., 2026b)	Tissue source, processing, retained ECM proteins, GAGs, architecture, stiffness, batch/source variability	High native complexity, but lower control and reproducibility
Biological context with usability	Natural and semi-synthetic hydrogels, including collagen, gelatin, HA/HAMA, fibrin, alginate, GelMA and hybrids (Tibbitt and Anseth, 2009; Alsharabasy and Pandit, 2024; Loessner et al., 2016; Bessot et al., 2025; Bessot et al., 2023; Kim et al., 2022; Cao et al., 2021; Ermis et al., 2023)	Adhesion, hydration, degradability, stiffness, porosity, gelation, culture format, stromal or niche phenotype	Balances biological context and practicality, but natural components do not automatically ensure physiological relevance
Mechanistic dissection of defined ECM cues	Synthetic or highly defined matrices, including PEG, PEG-4MAL, PVA, NorHA and peptide-functionalised hydrogels (Bock et al., 2023; Tibbitt and Anseth, 2009; Lin et al., 2023; Ligorio and Mata, 2023; Mosquera et al., 2022; Suwannakot et al., 2023; LeSavage et al., 2024; Kopyeva et al., 2025; Cruz-Acuña et al., 2023)	Ligand identity/density, protease-sensitive degradation, stiffness, viscoelasticity, stress relaxation, matched controls	Strong causal control, but reduced tissue-level complexity
Drug response/transport-sensitive endpoints	Spheroids, organoids or matrix-embedded models with defined diffusion environments (Xu et al., 2024; D’Angelo et al., 2026; Degerstedt et al., 2022; Druzhkova et al., 2022)	Porosity, permeability, gel density, diffusion distance, drug penetration, uptake, matrix-mediated resistance	Captures matrix-mediated drug response, but requires transport characterisation to distinguish diffusion limits from intrinsic sensitivity
Dynamic ECM/remodelling over time	Temporal-stiffening, viscoelastic, reversible-crosslink, degradable or self-healing hydrogels (Sinha et al., 2023; Lin et al., 2023; Major et al., 2024; Loebel et al., 2019; Tan et al., 2025; Khine et al., 2024; Feng et al., 2025)	Stress relaxation, creep, stiffness evolution, degradation, nascent ECM deposition, matrix recovery over culture time	Captures ECM evolution, but requires time-resolved characterisation

### 3.1 Models for tissue-specific fidelity and patient relevance

Tissue-derived ECM models are most useful when the biological question depends on native matrix complexity, tissue specificity or patient-relevant ECM context (Golebiowska et al., 2024). These include decellularised ECM (dECM) (Nam et al., 2024; Song et al., 2024; Hipwood et al., 2026b), cell-derived matrices (Barros da Silva et al., 2024), and basement membrane extracts such as Matrigel® (Nam et al., 2024; Song et al., 2024; Bhatia et al., 2026), which preserve structural and biochemical components of the native microenvironment to varying degrees (Hipwood et al., 2026a; D’Angelo et al., 2026; Golebiowska et al., 2024). Their strength is not that they reproduce all ECM features perfectly, but that they retain combinations of matrix proteins, growth factor-binding sites, glycosaminoglycans and tissue-specific cues that are difficult to reconstruct from individual components.

Several studies illustrate this use-case. In cervical squamous cell carcinoma, uterine cervical dECM supported lower initial organoid formation than Matrigel®, but supplementation with laminin-332 and laminin-111 improved organoid establishment. Importantly, uterine cervical dECM better preserved transcriptomic similarity to native tumour tissue and supported drug-response concordance with clinical patient response (Song et al., 2024). In colorectal cancer, intestinal dECM supported organoid growth similarly to Matrigel®, but promoted higher differentiation and pro-oncogenic marker expression, suggesting that tissue-matched dECM can shift organoid state toward more tissue-relevant phenotypes (Nam et al., 2024). In osteosarcoma organoids, matrix type and stiffness shaped invasion and doxorubicin response; lung dECM-MA revealed ECM-dependent invasion and drug response behaviours not reproduced by GelMA (Hipwood et al., 2026b). These examples support the use of dECM when preserving tissue-specific context or patient-relevant matrix response is central to the question.

The limitation is that the same complexity reduces control. dECM properties vary with tissue source, donor, processing and decellularisation efficiency, which can alter biochemical composition and mechanics (Golebiowska et al., 2024). Hybrid dECM approaches, including methacrylated dECM (Hipwood et al., 2026a; Dekker et al., 2026), and alginate- or PEG-containing blends (Milton et al., 2024; Barros da Silva et al., 2024), can improve handling, photocrosslinking, printability or mechanical robustness while retaining aspects of native biochemistry (D’Angelo et al., 2026; Milton et al., 2024; Barros da Silva et al., 2024). These approaches are useful when fidelity is needed but some standardisation or mechanical tuning is also required; however, they do not remove the need to characterise which matrix features are preserved and which are altered (Martinez‐garcia et al., 2021; Milton et al., 2024; Choi et al., 2025).

### 3.2 Models that balance biological context, usability and reproducibility

Natural and semi-synthetic matrices are useful when a model needs more biological context than a fully synthetic system, but greater tuneability and accessibility than dECM. This category includes collagen, gelatin, hyaluronic acid, fibrin, alginate, GelMA, HAMA and hybrid hydrogels (Tibbitt and Anseth, 2009; Alsharabasy and Pandit, 2024; Loessner et al., 2016). Their value lies in incorporating selected ECM features, such as adhesion, hydration, degradability or tissue-like mechanics, while retaining practical control over gelation, stiffness, culture format or biofabrication.

This intermediate role is useful for modelling tissue-inspired niches. Bessot et al. used GelMA-based systems to show how formulation and stiffness tuning can build specific prostate cancer niches (Bessot et al., 2025; Bessot et al., 2023). In one study, comparison of HAMA, GelMA and HAMA/GelMA identified 4% GelMA as the most adipogenic formulation, enabling adipocyte–prostate cancer co-cultures that revealed enzalutamide-associated lipid-metabolic dysregulation (Bessot et al., 2025). In a related model, tuning GelMA stiffness and culture conditions generated mineralised, adipose and tumour microtissues, with lower stiffness supporting larger prostate cancer spheroids and adipocyte-containing bone-like niches enhancing early tumour growth (Bessot et al., 2023). In breast cancer dormancy, comparisons across Matrigel®, collagen gels and PEG-RGD gels showed that long-term quiescence depends on both intrinsic and extrinsic cues, with the most dormant cells observed in 3D collagen gels (Kim et al., 2023). These examples show that natural and semi-synthetic matrices are valuable when modelling tissue-relevant states or multicellular niches while retaining experimental usability.

However, these systems should not be treated as automatically “physiological” because they contain natural components. Their relevance depends on whether selected ECM features match the tissue, disease context and endpoint being studied. For example, collagen–alginate matrices showed that storage modulus and network confinement can reversibly switch colorectal CAFs between inflammatory and myofibroblastic states through ROS–HIF-1α mechanotransduction (Cao et al., 2021). Similarly, HAMA/GelMA hydrogels recreating healthy, desmoplastic and hyperdesmoplastic pancreatic matrix stiffnesses showed that stiffer, hyperdesmoplastic matrix-mimicking gels increased proliferation-, EMT-, mechanotransduction- and progression-associated markers, including VIM and YAP, in pancreatic cancer–CAF spheroids (Ermis et al., 2023). Matrix properties can similarly regulate tumour-immune interactions. In 3D collagen-alginate cancer co-cultures, increasing stiffness promoted tumour-associated macrophage-like phenotypes, while in stiffness-tunable interpenetrating-network hydrogels, matrix mechanics and macrophages jointly regulated EMT-associated tumour-cell responses (Kuczek et al., 2019; Alonso-Nocelo et al., 2018; Guenther, 2024). Together, these studies show that semi-synthetic matrices are most informative when a tunable material property is linked to a defined stromal, immune or tumour phenotype, rather than invoked as a general marker of ECM mimicry.

### 3.3 Models for mechanistic dissection and defined ECM cues

Synthetic matrices are most useful when the aim is to isolate how defined ECM cues regulate cancer behaviour. PEG, PVA, NorHA and related hydrogels provide relatively inert platforms that can be functionalised with adhesive ligands, protease-sensitive crosslinkers or controlled mechanical and viscoelastic properties, enabling variables to be tuned more independently than in dECM or natural matrices (Tibbitt and Anseth, 2009; Ligorio and Mata, 2023).

Defined synthetic matrices first allow biochemical cues to be isolated. Bock et al. showed that PEG hydrogels presenting combined RGD, GFOGER and DYIGSR peptides enhanced metabolic activity of patient-derived breast cancer organoids compared with more limited ligand presentation (Bock et al., 2023). Katz and West used PEG hydrogels with variable integrin-binding and MMP-cleavable sites to show that epithelial tumours were more adhesion-responsive, whereas mesenchymal tumours were more stiffness-responsive (Katz and West, 2022). Mosquera et al. similarly found that PEG-4MAL hydrogels functionalised with GFOGER, REDV or RGD regulated prostate cancer organoid morphology, transcriptional/epigenetic state and inhibitor response (Mosquera et al., 2022).

Synthetic systems can also isolate mechanical and time-dependent cues. Bruns et al. showed that PEG-based single- and dual-stiffness hydrogels altered glioblastoma spheroid infiltration and chemotherapy response (Bruns et al., 2023), while Suwannakot et al. showed that PEG hydrogel stiffness and RGD presentation influenced MCF-7 spheroid formation and proliferation (Suwannakot et al., 2023). In stromal co-culture, Lin et al. used phenylboronic acid-based PEHNBA viscoelastic hydrogels to show that stress relaxation promoted CAF spreading and enhanced pancreatic cancer cell proliferation and EMT-associated secretory changes (Lin et al., 2023).

These examples show that synthetic matrices are not simply less biological; they are most informative when the hypothesis requires controlled perturbation of defined ECM cues to determine how specific matrix changes shape cancer phenotype or function. However, this control is achieved by simplifying tissue-level complexity, so synthetic matrices require careful justification of which ECM features are included, omitted or deliberately simplified for the biological question being tested.

## 4 Fit-for-purpose matrix characterisation

Across ECM-mimicking platforms, characterisation should be guided by the biological question rather than by a universal checklist. Broader materials-science frameworks have argued for systematic, multiscale and time-resolved ECM characterisation (Bock et al., 2024). In ECM-mimicking hydrogels specifically, this extends to less routinely reported properties such as porosity, viscoelasticity and permeability (Sui et al., 2025; Cameron et al., 2022). However, the practical challenge is to determine which of these properties are relevant to the specific model and endpoint. Micek et al. support this view by framing the tumour ECM as an evolving system in which composition, architecture and mechanics change together during progression, supporting engineered models that test defined ECM alterations rather than simply describing matrix complexity (Micek et al., 2020).

For mechanistic models, the tuned variables and controls should be explicit. LeSavage et al. addressed whether PDAC matrix stiffness drives chemoresistance, so they used defined HA–elastin-like protein matrices with tumour-matched stiffness while controlling HA and RGD content; high-stiffness matrices induced reversible chemoresistance in patient-derived PDAC organoids through CD44–hyaluronan-associated drug-efflux programmes (LeSavage et al., 2024). Kopyeva et al. asked how matrix mechanics and adhesive biochemistry regulate colorectal cancer growth and signalling, so they used PEG-based hydrogels with programmable stiffness and peptide presentation; soft matrices supported colony formation, whereas stiffer matrices restricted growth and collagen-derived DGEA altered EGF-induced signalling compared with fibronectin-derived RGDS (Kopyeva et al., 2025).

For drug-response models, the relevant descriptors shift toward transport. Degerstedt et al. addressed how ECM-like liver-tumour hydrogels affect doxorubicin diffusion, so they quantified diffusion in collagen/fibrin (ogen)-containing biomimetic gels and showed reduced diffusion coefficients compared with agarose (Degerstedt et al., 2022). Druzkhova et al. similarly examined how collagen architecture affects doxorubicin delivery and response, so they combined collagen-based 3D tumour models with SHG, multiphoton fluorescence and FLIM imaging, showing that collagen slowed doxorubicin diffusion, reduced cellular uptake and altered metabolic response (Druzhkova et al., 2022).

For immune-containing cancer models, matrix characterisation should be matched to the immune endpoint. In 3D immune and cancer-immune co-cultures, hydrogel formulation can alter T-cell and CAR-T cell activation (Revilla et al., 2025), high collagen density can suppress T-cell proliferation and cytotoxic function and is associated with reduced CD8^+^ T-cell infiltration (Kuczek et al., 2019), while increasing matrix stiffness can promote tumour-associated macrophage phenotypes and modify macrophage-dependent tumour cell invasion and EMT (Alonso-Nocelo et al., 2018; Guenther, 2024). Thus, fit-for-purpose characterisation does not require measuring every ECM property; it requires matching matrix descriptors to the biological process being interpreted.

## 5 Implementing ECM-informed model design

Once relevant ECM features have been defined, model design should deliberately perturb, reproduce or track them. A practical workflow is to define the expected ECM driver, select or engineer a matrix that varies that feature, and choose readouts that test the corresponding biological process. Cruz-Acuña et al. provide a clear example of this workflow. To examine how matrix mechanics contribute to oesophageal adenocarcinoma progression, they engineered NorHA hydrogels with constant RGD presentation and protease-dependent degradation but tunable stiffness, and selected readouts directly linked to tumour progression and mechanotransduction, including organoid formation, proliferation, YAP/SOX9 activation, CD44 expression and response to YAP inhibition (Cruz-Acuña et al., 2023).

This same logic can also be applied when the ECM feature of interest changes during culture. In this context, one priority would be to model ECM dynamics rather than only the starting or final matrix state. Major et al. used adipose dECM/silk fibroin hydrogels that stiffened during culture, allowing MCF-7 spheroids to experience progressive mechanical change. Temporal stiffening reduced E-cadherin, increased fibronectin, generated local spheroid heterogeneity and altered collagen I deposition, showing that programmed matrix evolution can reveal early breast cancer phenotypic transitions without complex stromal co-culture (Major et al., 2024).

A related challenge is that the matrix experienced by cells may not be limited to the input material. Nascent ECM deposition should therefore be considered as an emergent matrix feature during culture (Liu et al., 2026). Loebel et al. showed that cells in 3D hydrogels rapidly deposit pericellular matrix proteins that guide spreading, YAP/TAZ localisation and fate decisions (Loebel et al., 2019). Tan et al. further showed that hydrogel viscoelasticity regulates nascent ECM accumulation and remodelling (Tan et al., 2025). Thus, ECM-mimicking cancer models should be viewed as dynamic systems in which embedded cells respond not only to the input or starting material, but also to the nascent matrix they deposit during culture.

Dynamic and reversibly crosslinked hydrogels provide another example of fit-for-purpose implementation when the hypothesis involves time-dependent mechanics, multicellular assembly or matrix remodelling. Khine et al. used fast-relaxing RAFT polymer hydrogels with reversible boronate–diol bonding and dithiolane chemistry; in patient-derived pancreatic cancer cultures, fast relaxation induced a more mesenchymal phenotype, whereas dynamic stiffening restricted spheroid growth (Khine et al., 2024). Feng et al. used dual-dynamic polyglycerol hydrogels based on boronate bonds and Schiff-base interactions to provide tunable mechanics, support active cell remodelling and enable long-term spheroid formation across A549, HT-29, BT-474 and SK-BR-3 cells (Feng et al., 2025). Together, these studies show that reversible networks are particularly useful for testing time-dependent mechanics, multicellular assembly or matrix remodelling, provided stress relaxation, creep, stiffness evolution and ligand availability are characterised during culture. More broadly, these examples reinforce that progress will come not from adding complexity alone, but from designing matrices that test defined ECM mechanisms and linking matrix descriptors to biological outcomes.

## 6 Perspective: making ECM design explicit

The next stage of ECM-mimicking cancer models should not be defined by maximum complexity, but by clearer alignment between model purpose, matrix design and interpretation. Tissue-derived matrices are valuable when tissue specificity or patient relevance is central; natural and semi-synthetic matrices are useful when biological context must be balanced with usability; synthetic matrices are powerful when controlled perturbation of defined cues is needed; and dynamic systems are most informative when ECM evolution over time is part of the hypothesis. What unites these platforms is not their material composition but the design logic that should guide their use.

Progress will therefore depend on treating ECM variables with the same intentionality as cell source, genotype, culture medium or drug dose. This does not mean that every model should measure every matrix property. Instead, studies should define which ECM features are expected to influence the endpoint, report the matrix descriptors needed to interpret that endpoint, and acknowledge which features were simplified or omitted. Minimum reporting standard based on these principles would improve reproducibility and cross-material comparison. Studies that deliberately avoid confounded matrix conditions, or replace complex matrices such as Matrigel® with defined hydrogels to isolate mechanics, illustrate how simplification can become an explicit design choice rather than a hidden assumption (Hipwood et al., 2026a; Cruz-Acuña et al., 2023). For such standards to support model comparison, datasets should link matrix formulation and measured matrix descriptors, including stiffness, viscoelasticity, porosity, ligand density and remodelling capacity, to functional outcomes such as invasion, dormancy, stromal activation, immune cell infiltration and function, organoid growth or treatment response (Wu et al., 2026; Milton et al., 2024). Without this linkage, AI-assisted approaches (Jin et al., 2026), and materials-science frameworks (Bock et al., 2024), remain constrained, because matrix properties may be described without capturing their functional consequences.

Materials-science frameworks and AI-assisted approaches may improve model comparison and design, but only if datasets link matrix formulation and measured ECM properties to functional outcomes such as invasion, dormancy, stromal activation, organoid growth or treatment response.

Ultimately, ECM mimicry should move from a descriptive label to a hypothesis-driven design principle. The most useful ECM-mimicking cancer model is not necessarily the most complex one, but the one whose matrix features are deliberately chosen, clearly reported and interpreted in relation to the biological question being asked. If the ECM is treated as a central experimental variable rather than a background culture condition, the field stands to gain more interpretable data, more meaningful comparisons across platforms, and stronger mechanistic and translational conclusions from *in vitro* cancer systems.
